# Notes and News

**Published:** 1891-02-21

**Authors:** 


### Notes and News.

Collected and Communicated.

Scio House, Mrs. Scaramanga's generous gift to the poor of Shanklin, was opened on February 5th as a surgical home for children. Only invited guests were present at the opening ceremony, which was conducted by the Rev. C. T. Burland. The little hospital contains a ward with six beds, an operating-room, and excellent offices for the staff, and is fitted with every modern convenience, the general arrangements being such as to elicit warm approval from the medical men of the town.

The report of the Oldham Infirmary for 1890 shows that considerable progress has been made in the nursing department. A scheme has been drawn up and regulations adopted in accordance with which a surgeon requiring a nurse can apply to the Matron. The scale of charges is fixed and moderate, and the plan is found to work well. The Infirmary has been affiliated to the "National Pension Fund for Nurses," and a fund is to be formed to aid nurses to avail themselves of its benefits. The only part of the report which is not entirely satisfactory is the financial statement. In spite of care and economy, the adverse balance of 1889 has now been increased by £215 0s. 6d., making a debt on the hospital of £781 2s. 11d. The medical report states that 640 in-patients and 3,904 out-patients have been treated; of the former, 364 have been discharged as cured, and 160 as relieved.

A new departure has been entered upon at Sir Titus Salt's Hospital, Shipley. Since the election of a new Board of Governors, about five months ago, a number of meetings have been held between the Governors and the medical men of Shipley, with the object of formulating a new scheme of management so far as the medical staff is concerned, which should be agreeable alike to the Governors, the medical practitioners, and the public. As a result of these conferences and of inquiries made with regard to the management of other public hospitals, a scheme was drawn up by which it was arranged that the honorary medical staff should consist of all the duly qualified and registered medical men practising and residing within the townships of Shipley, Windhill, and Baildon. The honorary medical staff are empowered to appoint two persons from among themselves to take charge of the hospital for a period of three months at a time. No important surgical operation is to be undertaken without consultation between all the available members of the staff. The regulation which has aroused most interest and discussion is that with reference to out-patients. It is stipulated under this head that the hospital medical staff shall attend as out-patients only those who are already on the pensioners' list and those who are considered by the Governors to be proper candidates for pensions or temporary relief. In future, therefore, people who wish to become out-patients must first satisfy the Governors that they are suitable applicants for relief from the charity fund.

Mr. Richard Greene, F.R.C.P.E., will read a paper on "Hospitals for the Insane and Clinical Instruction in Asylums," before the Hospitals Association at the West-minster Hospital, Broad Sanctuary, on Wednesday, the 25th inst. The chair will be taken at eight p.m. by Mr. C. A. Lockhart Robertson, M.D., F.R.C.P. (Lord Chancellor's Visitor in Lunacy), and an interesting discussion is anticipated. Tickets may be procured on application to the Secretary of the Association, 140, Strand, W.C.

We learn that the Court of Governors of Guy's Hospital have acceded to the request of the staff for increased accommodation in the medical school. The proposed buildings comprise a dental department consisting of a conservation-room 110 ft. long and 24 ft. wide, a dental laboratory, and waiting-rooms for male and female patients. Adjoining the conservation-room will be a large lecture-room for dental lectures, and for those on experimental physics. Facing the conservation-room, but of lower level to avoid obstructing light, will be a new chemical laboratory to accommodate 100 students working at one time. The cost, including that of rebuilding the hospital workshops, is estimated at £10,000. The architects are Messrs. Woodd and Ainslie, who lately designed the Residential College.

The Alexandra Hospital for Children with Hip Disease is sadly in want of funds—a state of affairs which surely only requires to be known in order to be rectified. This hospital, which carries on its good work so unobtrusively, fills a much-felt want in London, where an appallingly large number of children suffer from this painful disease. The sixty beds in the Alexandra, and the twenty-one beds in the branch establishment at Bournemouth, are always filled. A charge of 4s. a-week is made for each child, but this does not nearly cover the cost of maintenance, to meet which the Committee depend upon annual subscriptions and donations, and these, during the last two years, have been insufficient. It is proposed that a larger and more suitable building be erected on the same site. To do this a sum of £10,000 is required.

The annual Court of the Royal Free Hospital, Gray's Inn Road, was held in the board-room, on the 12th inst., Mr. W. Tarn Pritchard, Chairman of the Committee, presiding. Amongst those present were: Mr. E. Masterman (Treasurer), Mr. Gainsford Bruce, M.P., Mr. C. Burt, Mr. Holroyd Chaplin, Mr. Acton G. Davis, Mr. J. Green, Mrs. Thorne (Hon. Secretary of the London School of Medicine for Women), Mrs. Lawrie, Mr. F. Ingle, Mr. E. Ford North (Chairman of the Weekly Board), Mr. F. D. Prideaux, Lieutenant-General Gordon Pritchard, C.B., Mr. H. Silver, and Mr. Conrad W. Thies (Secretary). The sixty-third annual report showed that during the past year 30,205 patients were admitted to treatment, viz., 2,138 in-patients, 17,263 out-patients, and 10,804 casualties. The ordinary receipts amounted to £6,048 17s., and legacies to £6,855 14s., making the total income £12,905, as against an ordinary expenditure of £10,672. The Chairman, in moving the adoption of the report, was glad to note that the amount of subscriptions again showed an increase, although the expenditure was rather larger than in previous years owing to the enhanced prices of many articles of ordinary consumption. The report was adopted, and votes of thanks accorded to the medical staff. An interesting feature of the proceedings was the presentation of an illuminated address to Mr. F. J. Gant, the Senior Surgeon, who has retired after rendering valuable services to the hospital for the past thirty-seven years.

